# 

**Published:** 1906-07-14

**Authors:** 


					rSnha/?rir*tin? Tormal
No. 1,031 VoT. XL. Satdbdat,
July 14th, 1906.
[Sabaoription.Tern-] PRICE THREEPENCE

				

